# Trajectories of perceived susceptibility to COVID-19 over a year: The COVID-19 & chronic conditions (C3) cohort study

**DOI:** 10.1097/MD.0000000000029376

**Published:** 2022-06-17

**Authors:** Lauren A. Opsasnick, Laura M. Curtis, Mary J. Kwasny, Rachel O’Conor, Guisselle A. Wismer, Julia Yoshino Benavente, Rebecca M. Lovett, Morgan R. Eifler, Andrea M. Zuleta, Stacy Cooper Bailey, Michael S. Wolf

**Affiliations:** a Department of General Internal Medicine, Northwestern University Feinberg School of Medicine, Chicago, IL; b Center for Applied Health Research on Aging (CHARA), Northwestern University Feinberg School of Medicine, Chicago, IL; c Department of Epidemiology, University of Michigan School of Public Health, Ann Arbor, MI; d Department of Preventive Medicine, Northwestern University Feinberg School of Medicine, Chicago, IL.

**Keywords:** Chicago, comorbidity, coronavirus disease 2019, coronavirus disease 2019 perceived susceptibility, socio-demographic health disparities, trajectories

## Abstract

The U.S. public health response to coronavirus disease 2019 (COVID-19) has been widely criticized as having downplayed the potential implications COVID-19 could have on one's personal health. Despite the unprecedented threat of COVID-19, many individuals still believed that it was not at all likely that they would become infected. We sought to investigate trends in adults’ perceived susceptibility to COVID-19 over the first year of the pandemic, whether distinct trajectories emerged, and if these trajectories differed by participant socio-demographic characteristics.

This was a longitudinal cohort study with 5 time points of data collection (March 13, 2020–March 3, 2021). Subjects included 627 adults living with ≥1 chronic conditions, who completed a baseline interview and at least one follow-up interview. In addition to collecting relevant socio-demographic characteristics, participants’ perceived susceptibility to COVID-19 across time was assessed and classified into distinct trajectories.

Nearly two-thirds (62.2%) of participants perceived themselves to be highly susceptible to COVID-19 from the onset of the pandemic (“early responders”) and sustained this over a year, a third (29.0%) eventually perceived themselves to be highly susceptible (“late responders”), and 8.8% maintained a low likelihood of susceptibility throughout the pandemic (“non-responders”). In multivariable analyses, compared to White participants, Latinx participants were significantly more likely to be non-responders and report low likelihood of perceived susceptibility (Risk Ratio [RR]: 3.46; 95% confidence interval: 1.19, 10.1), as were Black participants (RR: 5.49; 95% confidence interval: 2.19, 13.8).

A year into the COVID-19 pandemic, 1 out of 11 participants persistently did not think they might be susceptible and potentially infected. Future studies are needed to understand reasons why certain individuals, particularly those of racial/ethnic minorities, did not perceive themselves at risk for infection.

## 1. Introduction

The severe acute respiratory syndrome coronavirus 2 and resultant coronavirus disease 2019 (COVID-19) have evolved into an unprecedented pandemic, requiring communities to mobilize quickly to realize the threat and take action to prevent infection and spread.^[1]^ However, due to a public health response in the United States that has been widely recognized as misguided and fraught with misinformation, many adults may have been slow to realize the potential implications this coronavirus could have on their or a loved one's personal health.^[2]^

At the earliest onset of COVID-19's presence throughout the U.S., the COVID-19 & chronic conditions (C3) study was launched to investigate the knowledge, attitudes and actions related to COVID-19 among a cohort of middle age and older adults with one or more chronic conditions. We previously reported that in early March 2020, despite cases rapidly accelerating, a quarter of C3 participants reported believing that it was not at all likely that they would eventually get sick with the illness.^[3]^

As a follow-up, we sought to longitudinally examine the yearlong trend of adults’ perceived susceptibility to COVID-19, and determine whether some individuals followed similar progressions and could thereby be classified into distinct trajectories. Furthermore, we explored whether the trajectories differed by participant socio-demographic characteristics.

## 2. Methods

### 2.1. Study design

The C3 study is an on-going longitudinal, telephone-based survey that began at the onset of the COVID-19 pandemic. The initial survey was conducted from March 13 to 20, 2020 during the very first week of the outbreak in Chicago, Illinois. Over the course of a year, four subsequent study interviews, referred to as waves, occurred (Wave 2: March 27–April 3, Wave 3: May 1–May 20, Wave 4: July 15–August 18, Wave 5: November 30–March 3, 2021). The Illinois stay-at-home order began on March 20th, immediately after Wave 1 was completed. Chicago experienced a peak in COVID-19 cases in May, which resulted in an extension of the order through May 29, 2020. Waves 2 and 3 occurred during this period. Starting June 4, Chicago reached a plateau phase with the first surge, where the 7 days rolling average positivity rate was <10% and remained through Wave 4 data collection. Finally, during Wave 5, Chicago once again reached a peak in COVID-19 cases, where the positivity rate topped 13% during mid-November.^[4]^ The Northwestern Institutional review board approved all study procedures.

### 2.2. Study participants

Eligibility criteria included participants actively enrolled in one of five ongoing, National Institutes of Health (NIH)-funded research studies managed by our research team. All parent studies excluded individuals with severe hearing, vision and cognitive impairments due to concerns regarding the informed consent process. Four of the five research studies included only English-speaking subjects, while one study included Spanish-speaking subjects as well. The five parent studies have been previously described in our earlier publications, including full inclusion and exclusion criteria. These studies are comprised of mostly older adults with multiple chronic conditions. All participants have received medical care at one of five academic internal medicine practices or two federally qualified health centers throughout the greater Chicago metropolitan area.^[3,5,6]^

Trained research coordinators recruited participants from their parent studies to participate in a brief phone questionnaire pertaining to COVID-19. Survey data was collected using Research Election Data Capture (REDCap). Each survey averaged 20 to 40 minutes, and participants were compensated with a $10 to $15 gift card for their time, depending on the wave. A total of 673 participants were enrolled in the study and completed a Wave 1 interview. Of those enrolled, 626 participants completed a Wave 2 interview (93.3% cooperation rate), 601 completed a Wave 3 interview (89.7%), 558 completed a Wave 4 interview (82.9%), and 544 completed a Wave 5 interview (80.8%). For the purposes of this analysis, we included 627 participants who completed both their baseline survey, in addition to at least one follow-up survey.

### 2.3. Covariates and study outcomes

At all five waves, participants were surveyed about their perceived susceptibility to COVID-19, asking if they thought they would get sick from coronavirus (not at all, it's possible, I probably will, I definitely will). This outcome measure was dichotomized (not at all vs. it's possible, I probably will, I definitely will) to distinguish between participants who had any level of perceived susceptibly versus no susceptibility (yes/no).

Across all five NIH parent studies there was uniform data collection of participant demographics (age, sex, race/ethnicity, and language proficiency), socio-economic status (income and employment status), and self-reported number of chronic conditions. A single item captured participant self-reported overall health (excellent, very good, good, fair, and poor). All studies measured health literacy: four used the newest vital sign, and one used the validated brief health literacy screen single-item question, “*How confident are you filling out medical forms by yourself?*”’ Research has shown that the classifications from these instruments are highly correlated.^[7]^

### 2.4. Statistical methods

Descriptive statistics (means with standard deviations and percent frequencies) were calculated for all participant characteristics and survey responses. We then identified groups of individuals following similar progressions of perceived susceptibility to COVID-19 over multiple waves and classified them into trajectory groups using the *traj* command in Stata/SE, version 15 (StataCorp, College Station, TX, US).^[8]^ This method estimates discrete mixture models on longitudinal data, in our case assuming a Bernoulli distribution (logistic model) for the dichotomous perceived susceptibility variable. We used the Bayesian information criterion to determine the number of discrete trajectories in the data. Participants were assigned to a trajectory based on posterior probabilities of belonging to each group.^[9]^ Associations between participant characteristics and their assigned trajectory group were examined in bivariate analyses using chi-square tests. A multivariable Poisson model was used to estimate relative risks (with 95% confidence intervals (CIs)) of following a certain trajectory.^[10]^ Models adjusted for potential confounders (age, gender, race, income, health literacy, employment status, and primary care setting), as well as parent study. All statistical analyses were performed using Stata/SE, version 15 (StataCorp).

## 3. Results

Table 1 provides a summary of participant characteristics. The average age of participants was 63 years (mean: 62.8 standard deviation: 10.9). The majority were female (60.8%), 21.5% identified as Latinx, and 30.7% identified as Black. Nearly a third were living below the federal poverty level (28.8%) and 26.5% were working for pay. The majority of participants had three or more chronic conditions (63.0%) and a total of 22.3% and 22.8% had low or marginal health literacy, respectively.

**Table 1:** Participant characteristics.

Variable	Summary valueN = 627
Age, M (SD)	62.8 (10.9)
Age group, %	
<60	34.8
60–69	37.2
≥70	28.1
Female, %	60.8
Race, %	
Latinx	21.5
White	47.8
Black	30.7
Limited English proficiency, %	10.9
Living below poverty level, %	28.8
Employment status, %	
Working for pay	26.5
Not working (retired/unemployed)	73.5
Health literacy, %	
Low	22.3
Marginal	22.8
Adequate	54.9
Number of chronic conditions, %	
1	21.1
2	16.0
3 or more	63.0
Self-reported overall health, %	
Excellent	8.3
Very good	28.7
Good	39.7
Fair	19.8
Poor	3.5
Primary care setting, %	
AMC	70.2
FQHC	29.8
Parent study, %	
Study 1	18.8
Study 2	22.8
Study 3	32.1
Study 4	6.1
Study 5	20.3

AMC = Academic Medical Center, FQHC = Federally Qualified Health Center

**Figure 1:**
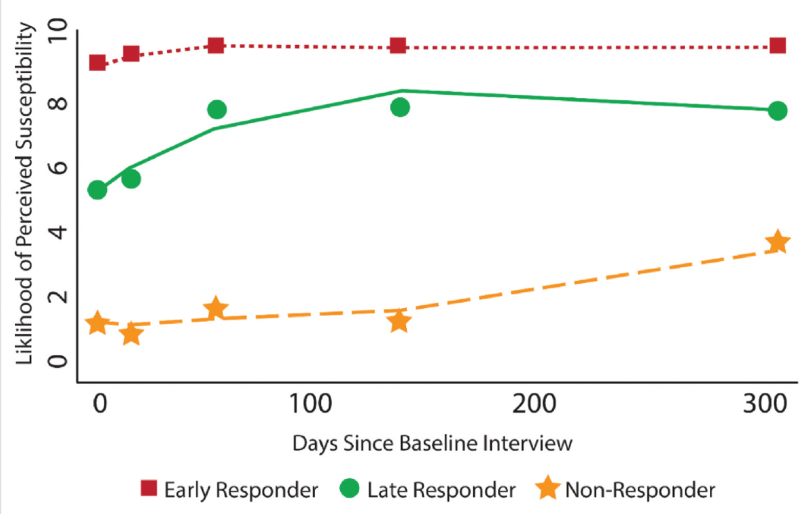
Trajectories of perceived susceptibility to COVID-19.

Participant responses varied across waves when asked if they thought they would get sick from coronavirus. Table S1, Supplemental Digital Content shows the full set of categorical responses. When dichotomized, 24.9% of participants had no perceived susceptibility to COVID-19 at Wave 1. This number continued to decline across the next four waves (22.5%, 13.8%, 13.5%, and 11.5%, respectively). When modeling the trajectory of perceived susceptibility to COVID-19, three distinct groups emerged, as shown in Figure 1. The first group (“early responders”) made up 62.2% of the sample and included participants who perceived themselves to be highly susceptible to COVID-19 from the onset of the pandemic. The second group (“late responders”) made up 29.0% of the sample and included participants who eventually perceived themselves to be highly susceptible. Finally, the third group (“non-responders”) made up 8.8% of the sample and included participants maintained a low likelihood of susceptibility throughout the pandemic.

In bivariate analyses, trajectory group was associated with several participant characteristics (Table 2). A greater proportion of early responders were White (57.5%) compared to Latinx (20.2%) or Black adults (22.3%), whereas non-responders were more likely to be Black (58.5%) than Latinx (28.3%) or White (13.2%) (*P* < .001). Additionally, non-responders were more likely than early or late responders to live below the poverty level (41.8% vs. 22.0% and 39.4%; *P* < .001), to receive medical care at federally qualified health centers (45.5% vs. 26.4% and 32.4%; *P* = .01), to be unemployed or retired (79.6% vs. 69.8% and 79.5%; *P* = .03), and to have limited health literacy (47.3% vs. 17.2% and 25.8%; *P* < .001).

**Table 2:** Bivariate analysis by trajectory group (N = 627).

Variable	Early responder(n = 390)	Late responder(n = 182)	Non-responder(n = 55)	*P*
Age group, %				
<60	36.2	33.0	30.9	
60–69	36.9	35.7	43.6	.67
≥70	26.9	31.3	25.5	
Gender, %				
Female	57.7	67.0	61.8	.10
Male	42.3	33.0	38.2	
Race, %				
Latinx	**20.2**	**22.2**	**28.3**	
White	**57.5**	**37.4**	**13.2**	**<.001**
Black	**22.3**	**40.4**	**58.5**	
Limited English proficiency, %				
Yes	9.5	12.1	16.4	.25
No	90.5	87.9	83.6	
Living below poverty level, %				
Yes	**22.0**	**39.4**	**41.8**	**<.001**
No	**78.0**	**60.6**	**58.2**	
Employment status, %				
Working for pay	**30.2**	**20.5**	**20.4**	**.03**
Not working	**69.8**	**79.5**	**79.6**	
Health literacy, %				
Low	**17.2**	**25.8**	**47.3**	
Marginal	**19.0**	**31.3**	**21.8**	**<.001**
Adequate	**63.8**	**42.9**	**30.9**	
Number of chronic conditions, %				
1–2	37.2	38.5	30.9	.59
3 or more	62.8	61.5	69.1	
Self-reported health, %				
Good – excellent	77.7	73.6	80.0	.47
Fair – poor	22.3	26.4	20.0	
Primary care setting, %				
AMC	**73.6**	**67.6**	**54.6**	**.01**
FQHC	**26.4**	**32.4**	**45.4**	
Parent study, %				
Study 1	20.5	15.4	18.2	
Study 2	23.3	22.0	21.8	
Study 3	30.0	36.3	32.7	.88
Study 4	6.2	5.5	7.3	
Study 5	20.0	20.9	20.0	

AMC = Academic Medical Center, FQHC = Federally Qualified Health Center Bolded values are significant at the *P* < 0.05 level.

In order to better understand those participants who maintained a low likelihood of susceptibility to COVID-19 throughout the pandemic, the early responders and late responders were combined and compared to the non-responders. In multivariable analyses adjusting for age, gender, race, income, health literacy, employment status, primary care setting, and parent study (Table 3), compared to White participants, Latinx participants were significantly more likely to be non-responders (Risk Ratio [RR]: 3.46; 95% CI: 1.19, 10.1), as were Black participants (RR: 5.49; 95% CI: 2.19, 13.8).

**Table 3:** Multivariable analysis modeling non-responders (N = 627).

	Non-responder
Variable	RR (95% CI)	*P*
Age group, %		
<60	REF	–
60–69	1.49 (0.74, 2.99)	.27
≥70	1.49 (0.62, 3.59)	.38
Gender, %		
Male	REF	–
Female	0.93 (0.50, 1.71)	.80
Race, %		
Latinx	**3.46 (1.19, 10.1)**	**.02**
White	REF	–
Black	**5.49 (2.19, 13.8)**	**<.001**
Living below poverty level, %		
No	REF	–
Yes	1.01 (0.53, 1.96)	.97
Health literacy, %		
Low	1.95 (0.90, 4.21)	.09
Marginal	1.18 (0.52, 2.66)	.69
Adequate	REF	–
Employment status		
Not working	REF	
Working for pay	0.96 (0.47, 1.95)	.91
Primary care setting, %		
AMC	REF	–
FQHC	2.33 (0.72, 7.58)	.16
Parent study, %		
Study 1	REF	–
Study 2	0.26 (0.07, 1.00)	.05
Study 3	0.31 (0.08, 1.18)	.09
Study 4	1.17 (0.27, 5.05)	.83
Study 5	0.52 (0.20, 1.35)	.18

Bolded values are significant at the *P* < 0.05 level.

## 4. Discussion

Among this sample of middle age and older adults with at least one chronic condition, we identified three distinct groups related to perceived susceptibility to COVID-19 over the first year of the pandemic. These trajectories included those who always felt they were vulnerable to the virus, those who developed a belief of susceptibility over time, and those who have not felt vulnerable throughout the pandemic. While the majority of individuals recognized their potential to become infected by COVID-19, 1 out of 11 participants persistently did not recognize the threat. Given the entire C3 sample is considered at highest risk for adverse outcomes if infected, this is a concerning prevalence. Nonetheless, these findings are understandable, given the inconsistent messaging and the amount of misinformation surrounding the pandemic.

Our finding of disparities by race and ethnicity with regard to perceived susceptibility is in line with findings from a previous study by Scarinci et al,^[11]^ One possible explanation could be that Black and Latinx adults in our sample did not believe they were going to become infected with COVID-19 because they were taking the necessary public health actions to protect themselves and others. This includes social distancing, wearing a mask, and towards the latter end of data collection, even vaccination. All of these actions might have given confidence to individuals that they could avoid infection. Another explanation, presented by Plough et al, suggested inadequate knowledge of perceived risk of COVID-19 might drive these inequities.^[12]^ However, our team has previously published findings from this cohort that did not find evidence of racial or ethnic disparities in COVID-related knowledge.^[3]^

Prior research also has posited that cultural and/or spiritual differences, not assessed in our investigation, might explain differences in how one perceives an imminent health threat and an ability to influence the outcome. Similarly, Scarinci et al suggested that disparities in perceived vulnerability to COVID-19 might be driven by a sense of fatalism caused by longstanding mistrust in the government.^[11]^ It is possible that longstanding inequalities in socioeconomic status and life circumstances, racism, and mistrust in government might lead to a lost sense of ability to protect oneself from the coronavirus. Given that communities of color have been disproportionately affected by the pandemic in terms of both rates of infection and deaths, especially in Chicago, this could in part explain our findings.^[13,14]^ Additional qualitative studies are warranted to better understand these results.

There are certain limitations that should be noted. This study was comprised of participants from NIH-funded research that took place in one large U.S. city. Therefore, these findings may have limited generalizability, specifically for younger adults with no pre-existing conditions or those in rural settings. However, our sample was well characterized in terms of demographic and psychosocial factors. Additionally, one of the parent studies (chronic obstructive pulmonary disease) measured health literacy using the validated brief health literacy screen single item, rather than the Newest Vital Sign. However, sensitivity analyses excluding them from the analysis indicated that results were similar (results not reported). Finally, this analysis studied perceived susceptibility to COVID-19 using a single survey item that may or may not have had implications on any pandemic-related behaviors or outcomes.

Future qualitative studies might better elucidate the reasons why certain individuals, in particular those of racial/ethnic minorities, did not perceive themselves to be susceptible to the global threat of COVID-19, even as the pandemic progressed and racial disparities in infection and morbidity/mortality emerged. The pandemic has occurred during parallel, historical political and social movements in the U.S. that might have also influenced how one might perceive a public health threat in lieu of other threats of equal or even greater perceived significance. The link between beliefs about the coronavirus and public health actions and health outcomes should also be examined to further understand the ramifications of not acknowledging the personal risk of COVID-19.

## Author contributions

**Conceptualization:** Guisselle Wismer, Michael Wolf, Rebecca Lovett, Stacy Bailey

**Data curation:** Laura Curtis, Rebecca Lovett

**Formal analysis:** Laura Curtis, Lauren Opsasnick, Mary Kwasny, Rachel O’Conor

**Funding acquisition:** Michael Wolf, Stacy Bailey

**Investigation:** Laura Curtis, Michael Wolf, Stacy Bailey

**Methodology:** Guisselle Wismer, Laura Curtis, Lauren Opsasnick, Michael Wolf, Rachel O’Conor, Stacy Bailey

**Project administration:** Andrea Zuleta, Guisselle Wismer, Julia Benavente, Michael Wolf, Morgan Eifler, Rachel O’Conor, Stacy Bailey

**Resources:** Guisselle Wismer, Julia Benavente, Michael Wolf

**Software:** Lauren Opsasnick

**Supervision:** Laura Curtis, Lauren Opsasnick

**Visualization:** Guisselle Wismer, Julia Benavente, Stacy Bailey

**Writing – original draft:** Lauren Opsasnick

**Writing – review & editing:** Andrea Zuleta, Guisselle Wismer, Julia Benavente, Laura Curtis, Lauren Opsasnick, Mary Kwasny, Michael Wolf, Morgan Eifler, Rachel O’Conor, Rebecca Lovett, Stacy Bailey

## Supplementary Material

Supplemental Digital Content
