# Supplementary material for: Trajectories of perceived susceptibility to COVID-19 over a year: The COVID-19 & chronic conditions (C3) cohort study
Source: Medicine (Baltimore). 2022 Jun 17;101(24):e29376. doi: 10.1097/MD.0000000000029376 (PMC9276380; doi:10.1097/MD.0000000000029376)
Supplement: Supplemental Digital Content [file medi-101-e29376-s001.docx]

**Supplement Table 1. Perceived Susceptibility Variable across Waves**

|  | | **Wave 1**  (N=627) | **Wave 2**  (N=601) | **Wave 3**  (N=552) | **Wave 4**  (N=503) | **Wave 5**  (N=452) |
| --- | --- | --- | --- | --- | --- | --- |
| Do you think that you will get sick from the coronavirus? | |  |  |  |  |  |
|  | Not at all | 24.9 | 22.5 | 13.8 | 13.5 | 11.5 |
|  | It is possible | 65.4 | 64.4 | 65.4 | 60.4 | 59.7 |
|  | I probably will | 8.5 | 12.5 | 16.5 | 17.7 | 18.6 |
| I definitely will | | 1.3 | 0.7 | 4.4 | 8.4 | 10.2 |
